# Matrix Metalloproteinase-10/TIMP-2 Structure and Analyses Define Conserved Core Interactions and Diverse Exosite Interactions in MMP/TIMP Complexes

**DOI:** 10.1371/journal.pone.0075836

**Published:** 2013-09-20

**Authors:** Jyotica Batra, Alexei S. Soares, Christine Mehner, Evette S. Radisky

**Affiliations:** 1 Department of Cancer Biology, Mayo Clinic Cancer Center, Jacksonville, Florida, United States of America; 2 Biology Department, Brookhaven National Laboratory, Upton, New York, United States of America; Griffith University, Australia

## Abstract

Matrix metalloproteinases (MMPs) play central roles in vertebrate tissue development, remodeling, and repair. The endogenous tissue inhibitors of metalloproteinases (TIMPs) regulate proteolytic activity by binding tightly to the MMP active site. While each of the four TIMPs can inhibit most MMPs, binding data reveal tremendous heterogeneity in affinities of different TIMP/MMP pairs, and the structural features that differentiate stronger from weaker complexes are poorly understood. Here we report the crystal structure of the comparatively weakly bound human MMP-10/TIMP-2 complex at 2.1 Å resolution. Comparison with previously reported structures of MMP-3/TIMP-1, MT1-MMP/TIMP-2, MMP-13/TIMP-2, and MMP-10/TIMP-1 complexes offers insights into the structural basis of binding selectivity. Our analyses identify a group of highly conserved contacts at the heart of MMP/TIMP complexes that define the conserved mechanism of inhibition, as well as a second category of diverse adventitious contacts at the periphery of the interfaces. The AB loop of the TIMP N-terminal domain and the contact loops of the TIMP C-terminal domain form highly variable peripheral contacts that can be considered as separate exosite interactions. In some complexes these exosite contacts are extensive, while in other complexes the AB loop or C-terminal domain contacts are greatly reduced and appear to contribute little to complex stability. Our data suggest that exosite interactions can enhance MMP/TIMP binding, although in the relatively weakly bound MMP-10/TIMP-2 complex they are not well optimized to do so. Formation of highly variable exosite interactions may provide a general mechanism by which TIMPs are fine-tuned for distinct regulatory roles in biology.

## Introduction

The matrix metalloproteinases (MMPs) are a large family of secreted and membrane associated zinc-dependent endopeptidases with key roles in extracellular matrix remodeling. They are instrumental in regulation of cell growth, motility, tissue morphogenesis and response to injury, not only by degrading matrix proteins, but also via limited proteolysis of specific extracellular targets including growth factors, cytokines, receptors, and adhesion molecules [1], [2]. MMP proteolytic activity is regulated at multiple levels. MMPs are produced as zymogens requiring activation by other proteases [3], [4], and once activated, proteolytic activity is further regulated by a family of endogenous inhibitors, the tissue inhibitors of metalloproteinases (TIMPs) [5], [6]. Dysregulation and excessive activity of MMPs has been associated with many pathologies including arthritis, atherosclerosis, and cancer [1], [7]. MMP-10, also known as stromelysin-2, is capable of degrading a broad spectrum of extracellular matrix proteins [8], and of activating MMP-1, -7, -8, and -9 [9]. It appears to have distinct functions in cell migration during wound healing [10], [11], in bone development [12], and in vascular remodeling [13], [14]. MMP-10 has drawn interest as a potential therapeutic target, as it has been found to contribute to tumor growth and progression in cancers including non-small cell lung carcinoma [15], [16], [17], [18], head and neck cancer [19], and lymphoma [20].

TIMP-2 is one of a family of four mammalian protein protease inhibitors that inhibit MMPs, and in some cases the related disintegrin metalloproteinases (ADAMs) and disintegrin metalloproteinases with thrombospondin motifs (ADAM-TSs), in a 1∶1 stoichiometric fashion [5], [6]. The TIMPs have overlapping inhibitory specificity, and TIMP-2 has been reported to inhibit all MMPs that have been evaluated [5], [6], listed in the MEROPS database (http://merops.sanger.ac.uk/) [21] to include MMP-1, -2, -3, -7, -8, -9, -10, -13, -19, MT1-MMP, MT2-MMP, MT3-MMP, MT4-MMP, and MT6-MMP, as well as ADAM12 [22]. Inhibition constants (*K*_i_) for these interactions vary across more than six orders of magnitude, from 0.6 fM for full-length MMP-2 [23], to 5.8 nM for the MMP-10 catalytic domain [24]. Under *in vivo* conditions in which MMPs may be present in excess of TIMPs, it is anticipated that differential TIMP affinities will help to determine which MMPs are more likely to remain free and active. TIMPs are also multifunctional proteins with pleiotropic activities mediated through protein-protein interactions with other binding partners. In particular, TIMP-2 can associate with α3β1 integrin and consequently regulate cell cycle progression and angiogenesis via MMP-independent mechanisms [5], [6], [25], although the structural basis of this interaction is not yet well defined.

The general structural basis for inhibition of MMPs by TIMPs was revealed in crystal structures of the MMP-3/TIMP-1 [26] and MT1-MMP/TIMP-2 [27] complexes, and subsequently expanded with later structures of the MMP-13/TIMP-2 [28] and MMP-10/TIMP-1 [24] complexes, along with complexes of MMP-1 and MT1-MMP with the N-terminal domain of TIMP-1, which makes the majority of intermolecular contacts [29], [30]. However, to better understand the structural basis for TIMP function and specificity *in vivo*, we need structural examples not only of the strongest MMP/TIMP complexes, but of the full spectrum of possible interactions. Additionally, by elucidating the structural underpinnings of kinetic and thermodynamic discrimination in the association of natural TIMPs with MMPs, we will also gain insights to enable development of recombinant TIMP-based proteins with altered molecular selectivity.

Here, we report the structure of the human MMP-10 catalytic domain (MMP-10cd) bound to human TIMP-2. We previously have found that the interaction of these two proteins does not follow the slow, tight binding behavior typical of a majority of MMP/TIMP complexes [31], but rather follows a classic competitive inhibition model with a *K_i_* of 5.8 nM [24]. This new structure allows comparison with our previous crystal structure of the MMP-10cd/TIMP-1 complex to assess the extent to which the MMP-10cd adapts differently to each TIMP, and to relate differences in contacts at the molecular interfaces to the observed differential affinities of the complexes. We also make comparisons with the previously reported structures of three other MMP/TIMP complexes (MMP-3/TIMP-1 [26], MT1-MMP/TIMP-2 [27], and MMP-13/TIMP-2 [28]), enabling us to distinguish key conserved features of the inhibitory interface, as well as highly diverse contacts that vary widely among MMP/TIMP pairs and are anticipated to contribute to specificity *in vivo*.

## Materials and Methods

### Protein expression and purification

The recombinant human proMMP-10cd was expressed in *E. coli* strain BL21 (DE3), purified by Q-sepharose chromatography, refolded, activated and immediately purified to homogeneity by gel filtration on a Superdex 75 column equilibrated with 20 mM Tris-HCl, pH 7.0, 4 mM CaCl_2_ and 150 mM NaCl as previously described [24]. The recombinant human TIMP-2 was produced in HEK 293E cells and purified to homogeneity by Q-sepharose chromatography followed by gel filtration on Superdex 75 in 20 mM Tris, pH 8.5 and 150 mM NaCl as previously described [24].

### Crystallization and data collection

Purified active MMP-10cd and TIMP-2 were combined in 1∶1.1 (mol/mol) ratio and concentrated to a final concentration of 4.5–5.5 mg/mL. The initial crystallization screens were done using the hanging drop vapor diffusion method. Crystals of diffraction quality were grown at room temperature from droplets containing 0.1 M HEPES, pH 7.5, and 25% (w/v) PEG 2000 monomethyl ether. The crystals appeared in clusters and grew over the course of 1–2 weeks to 0.2×0.1×0.1 mm. Crystals were soaked in a cryoprotectant solution (0.1 M HEPES, pH 7.5, 25% (w/v) PEG 2000 monomethyl ether and 20% glycerol) and cryocooled in liquid N_2_.

Crystals were screened at beam line X29 of the National Synchrotron Light Source, Brookhaven National Laboratory. Data were collected at 100 K from one crystal that diffracted to 2.1 Å resolution. Crystals of MMP-10/TIMP-2 belong to the orthorhombic space group P1, with unit cell dimensions *a* = 37.69, *b* = 56.93, *c* = 82.59 and contain two copies of the heterodimeric complex in the asymmetric unit. The x-ray data were merged and scaled using DENZO/SCALEPACK [32].

### Structure determination

The x-ray structure of MMP-10cd in complex with TIMP-2 was solved by molecular replacement using Phaser [33] supported by CCP4. The structures of MMP-10cd from the MMP-10cd/TIMP-1 complex (PDB ID: 3V96, chain B) [24] and of full-length TIMP-2 (PDB ID: 1BR9) [34] were used as search models. A test set of 5% of the total reflections was excluded from refinement. Manual rebuilding was done in COOT [35]. Refmac5 [36] was used to carry out refinement and water molecules were added into difference peaks (*F_o_*-*F_c_*) greater than 2σ. Because of the presence of two copies of the complex in the asymmetric unit, non-crystallographic symmetry restraints [37] were employed to refine structures. NCS tight restraints were added individually to full-length chains of MMP-10cd and TIMP-2. Ultimately, the final steps of refinement were done in Refmac5 (5.7.0029) where automatically generated local NCS restraints were used to reduce the *R_free_* value from 29.6% to 26.3%. The final stage of refinement included addition of solvent molecules into peaks greater than 1σ and within acceptable H-bonding distance from neighboring protein atoms. The final model included 160 water molecules and gave *R_cryst_* (*R_free_*) of 0.216 (0.263). The quality of the final model was analyzed using MolProbity [38]. The Ramachandran plot for the two heterodimeric complexes in the asymmetric unit reveals 93.18% of all residues in the most favored regions, 98.07% in allowed regions and 13 residues as outliers: TIMP-2 residues Asn-33, Asp-34, Gly-79, Gly-80, Lys-81, His-120, and Asp-172 in chain A and Gly-32, Asp-34, Pro-56, Lys-81, and His-120 in chain B, as well as MMP-10cd residue Asn-240 in chain D. TIMP-2 residues 120 and 172 were previously found to be outliers in the crystal structure of unbound TIMP-2 (PDB ID: 1BR9) [34]. The coordinates and structure factors have been submitted to the RCSB Protein Data Bank under the accession code 4ILW.

Structure superpositions of MMP-TIMP complexes were performed using PYMOL (Python-enhanced molecular graphics tool). Structure figures were created using PYMOL. Interface analyses of accessible surface area and changes in solvation energy were performed using the PISA server (PDBePISA Protein Interfaces, Surfaces and Assemblies) [39].

## Results and Discussion

### Overall structure of the MMP-10cd/TIMP-2 complex

The structure of human MMP-10cd bound to human TIMP-2 was determined at 2.1 Å resolution in space group P1 with two complexes per asymmetric unit. The structure was solved by molecular replacement, using as search models the previously reported structures of MMP-10cd from the MMP-10/TIMP-1 complex (PDB ID: 3V96, chain B) [24] and unbound full-length TIMP-2 (PDB ID: 1BR9) [34] as described in “Materials and Methods”. The model was refined to a final *R_cryst_* (*R_free_*) of 21.6% (26.3%). The final model included 341 protein residues in each of the two complexes, with three calcium ions and two zinc ions bound per MMP-10 molecule, and possessed a total of 160 water molecules. The crystallographic statistics are summarized in Table 1. The two inhibitor/enzyme complexes in our structure (molecules A/D and B/F) showed small but noticeable structural deviations throughout, and alignment of the two complexes yielded an r.m.s.d. for all equivalent C^α^ atoms of 1.150 Å. The two complexes differed significantly in the quality of electron density maps throughout several partially disordered regions, and these regions account for most of the differences contributing to the overall r.m.s.d. Specifically, regions of poorer electron density for the second TIMP-2 molecule (chain B) include AB loop residues 30–41, the connector-D loop residues 78–83, GH loop residues 132–143, and the loop connecting helices H4a–H4b (residues 151–157). For the purposes of representation in figures, comparison with other MMP/TIMP structures, and subsequent discussion, we have used as our point of reference the better-ordered structure of the complex formed between molecules A and D.

**Table 1:** Crystallographic data collection and refinement statistics for MMP-10cd/TIMP-2 complex.

PDB ID	4ILW
Complexes per ASU	2
Space group	P1
Unit cell, Å	37.69, 56.93, 82.59 76.14°, 79.84°, 71.25°
Resolution, Å	2.1
Unique reflections	35606
Completeness, %	97.66 (94.0)^a^
Multiplicity	3.1 (2.6)^a^
Mean I/(σ)	11.2 (2.2)^a^
*R*_-merge_	0.073 (0.415)^a^
Wilson B-factor, Å^2^	38.2
R_cryst_/R_free_ (%)	0.216/0.263
Average B-factor, Å^2^	41.4
Protein atoms	5378
Water molecules	160
R.m.s.d. bonds, Å	0.014
R.m.s.d. angles, °	1.667
Φ,Ψ angle distribution^b^ In favored regions	628 (93.18%)
In allowed regions	661 (98.07%)
Outliers	13 (1.93%)

a Values in parentheses are for the highest resolution shell (2.14–2.1 Å).

b Ramachandran distribution is reported as defined by Molprobity/PDB validation.

**Figure 1:**
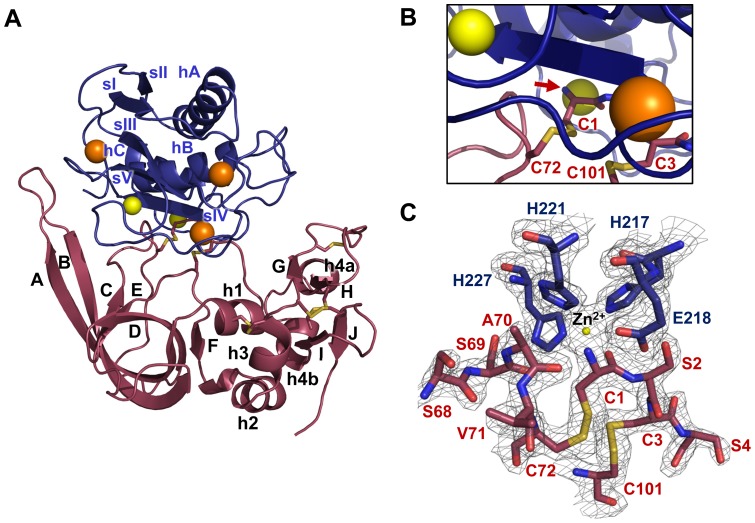
Crystal structure of MMP-10cd/TIMP-2 complex. (A) Structural overview of the complex shows the MMP-10cd in blue, zinc ions in yellow, calcium ions in orange, disulfide bridges in gold, and TIMP-2 in raspberry. The N-terminal domain of TIMP-2 is positioned to the left, comprising β-strands A–F, helices 1–3 and intervening loops, and the C-terminal domain is positioned on the right, comprising β-strands G–J, helices 4a and 4b, and connecting loops. Numbering shown is consistent with early descriptions of TIMP-1 and TIMP-2 structures from the Bode group [26], [27]; helix 4a within the multiple turn loop was originally not numbered. (B) A closer view of the cartoon structure in the vicinity of the active site shows the TIMP-2 N-terminal residue Cys-1 (red arrow) coordinated to the catalytic zinc ion directly behind. (C) The 2F_o_-F_c_ electron density map contoured at 2.0σ around the MMP-10cd active site shows the catalytic zinc coordinated by side chains of His-217, Glu-218, His-221, and His-227. The bound molecule of TIMP-2 also coordinates the catalytic zinc via the terminal amine and carbonyl oxygen of Cys-1. Numbers shown in blue correspond to MMP-10 residues and numbers in red to TIMP-2 residues.

Globally, the MMP-10cd/TIMP-2 complex resembles other structures of MMP catalytic domains that have been solved in complex with TIMPs (**Fig. 1a**). The 159-residue MMP-10cd displays the classic metzincin fold [40] as found previously [24], [41]. The fold consists of three α-helices, four parallel and one antiparallel β-strands, and several long connecting loops, and is stabilized by three Ca^2+^ and two Zn^2+^ ions. The 182-residue TIMP-2 is comprised of two domains and is stabilized by six disulfide bridges, as previously described [34], [42]. The larger N-terminal domain, primarily responsible for MMP inhibitory activity, possesses the oligosaccharide/oligonucleotide binding (OB) fold, characterized by a five-stranded β-barrel. The smaller C-terminal domain, which can mediate binding to the hemopexin domain of MMPs including MMP-2 [43], [44], [45], is comprised of two pairs of β-strands and several short helical segments connected by loops. The ridge of TIMP-2 formed by the inhibitor N-terminal segment linked by disulfide bonds to the C-connector and EF loops fills the MMP-10 substrate-binding cleft, while the long AB loop and several residues from the C-terminal domain of TIMP-2 form additional contacts with the MMP-10cd (**Fig. 1a**). In the MMP-10 active site, where the catalytic zinc ion is coordinated by residues of the HE*XX*H*XX*G*XX*H motif, the N-terminal amine of TIMP-2 Cys-1 directly coordinates to the catalytic zinc (**Fig. 1b, 1c**), consistent with the conserved mechanism of inhibition seen in complexes of MMPs with human TIMP-1 [**24**], [**26**] and with bovine TIMP-2 [27], [28].

### Structural changes induced by complex formation

**Figure 2:**
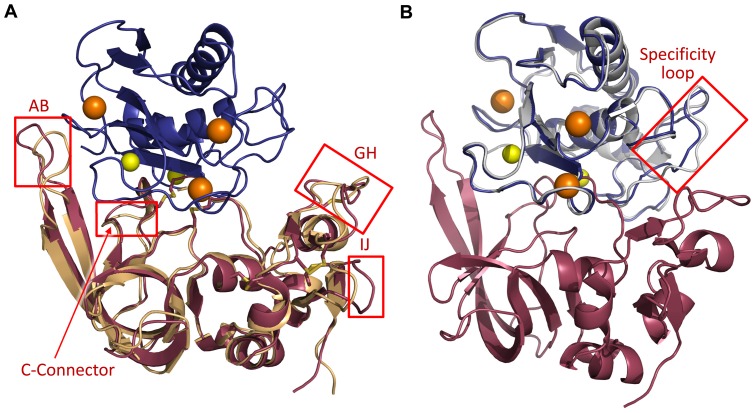
Structural adaptations induced by MMP-10cd/TIMP-2 association. (A) Structural differences in TIMP-2 bound to the MMP-10cd relative to the crystal structure of unbound TIMP-2 (PDB ID: 1BR9 [34], shown in wheat) are confined to several surface loops, most notably the AB, C-connector-D, GH, and IJ loops, highlighted by red boxes. (B) Structural alterations in MMP-10cd relative to the crystal structure of MMP-10cd bound to small molecule inhibitor NNGH (PDB ID: 1Q3A [41], shown in white) are limited to the specificity loop, highlighted by the red box. Superpositions are based on the C_α_ atoms of all corresponding residues.

In comparisons of the MMP-10/TIMP-2 complex (colored indigo/raspberry in **Fig. 2**) with the free TIMP-2 structure (colored wheat in **Fig. 2A**; PDB ID: 1BR9) [34], significant deviations observed in several flexible loops suggest structural accommodations made by the inhibitor upon MMP-10 binding. The overall r.m.s.d of all equivalent C^α^ atoms is 0.945 Å, but major differences are confined to a few loops. In particular, large backbone conformational changes are found in the AB loop, C-connector loop, connector-D loop (obscured in the figure), GH loop, and IJ loop (**Fig. 2a**). Changes in the AB loop, C-connector loop, and GH loop conformations serve to optimize specific protein-protein contacts at the enzyme-inhibitor interface, described below under “*Contacts at the MMP-10cd/TIMP-2 complex interface*”. Alterations in the connector-D and IJ loops are more likely influenced by nearby crystal contacts, since these loops are located distant from the interface with the MMP-10cd.

Comparing the MMP-10cd/TIMP-2 complex with the structure of the MMP-10cd bound to small molecule inhibitor *N*-isobutyl-*N*-[4-methoxyphenylsulfonyl]glycyl hydroxamic acid (NNGH) (colored white in **Fig. 2B**; PDB ID: 1Q3A) [41], we find a lesser degree of structural alteration for MMP-10 upon complex formation, with an overall r.m.s.d of equivalent C^α^ atoms of 0.422 Å. Major structural differences are confined to MMP-10 specificity loop residues 239−249 (**Fig. 2b**). This highly flexible loop is involved in shaping the hydrophobic S_1_′ subsite, a major determinant of substrate specificity [40]. In the NNGH-bound structure, residues 242−244 in two of three molecules in the asymmetric unit are unstructured and absent in the model. In the MMP-10cd/TIMP-2 complex, this stretch of residues was ordered enough to include in our model, but high B-factors and weak electron density throughout the loop hint at remaining conformational heterogeneity.

### Contacts at the MMP-10cd/TIMP-2 complex interface

**Figure 3:**
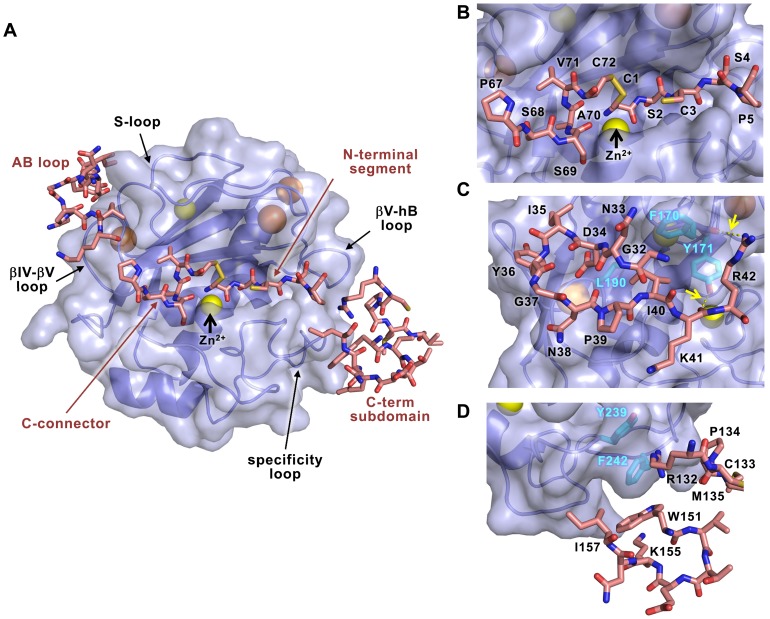
Contacts at the MMP-10cd/TIMP-2 interface. MMP-10cd is rendered as a cartoon covered by semitransparent surface (slate) in the standard frontal orientation, with horizontally aligned TIMP-2 segments in stick representation (salmon). (A) Overview shows contacts of the TIMP-2 C-connector and N-terminal segment with MMP-10 substrate binding cleft (center), TIMP-2 AB loop contacts with MMP-10 S-loop and βIV-βV loop (upper left), and TIMP-2 C-terminal domain contacts with MMP-10 specificity and βV-hB loops (lower right). (B) Closer view of MMP-10 substrate binding cleft shows TIMP-2 C-connector residues occupying nonprimed subsites to the left of the catalytic zinc, while TIMP-2 N-terminal residues occupy primed subsites to the right of the zinc. (C) Closer view of the AB loop interactions reveals two interfacial H-bonds (dotted yellow lines highlighted by yellow arrows), and burial of the Ile-40 side chain in a hydrophobic pocket formed by MMP-10 residues Phe-170, Tyr-171, and Leu190. (D) GH loop residues 132–135 and multiple turn loop residues 151–157 on the C-terminal domain of TIMP-2 form minimal interactions with the MMP-10cd, including ring-stacking and cation-π interactions with MMP-10 specificity loop residues Phe-242 and Tyr-239.

The MMP-10cd is contacted by an extended surface of TIMP-2 formed by multiple segments of the inhibitor N- and C-terminal domains, including the N-terminal segment, C-connector, AB loop, and several loops of the C-terminal domain (**Fig. 3a**). In total, the interaction involves 35 residues of MMP-10 and 39 residues of TIMP-2, and results in burial of 1247 Å^2^ of accessible surface area on the MMP-10cd and 1232 Å^2^ of accessible surface area on TIMP-2. The MMP-10cd/TIMP-2 complex interface features two salt bridges, one of which fulfills H-bond criteria, and 18 additional interfacial H-bonds as summarized in **Table 2**.

**Table 2:** MMP-10cd/TIMP-2 interface electrostatic interactions.

MMP-10		TIMP-2			
residue	atom	residue	atom	distance	type*
His-217	[NE2]	Cys-1	[O]	2.79	H
His-227	[NE2]	Cys-1	[O]	3.13	H
Glu-218	[OE1]	Cys-1	[O]	3.34	S
Glu-218	[OE2]	Cys-1	[O]	2.95	H,S
Leu-180	[N]	Ser-2	[O]	2.67	H
Ala-181	[N]	Ser-2	[O]	3.61	H
Ala-181	[O]	Ser-2	[N]	3.15	H
Glu-218	[OE1]	Ser-2	[N]	3.03	H
Glu-218	[OE1]	Ser-2	[OG]	2.53	H
Pro-237	[O]	Cys-3	[N]	2.93	H
His-178	[O]	Ser-4	[N]	2.83	H
His-178	[N]	Ser-4	[OG]	3.63	H
Tyr-191	[OH]	Pro-39	[O]	3.43	H
Tyr-171	[OH]	Lys-41	[O]	3.00	H
Phe-170	[O]	Arg-42	[NH1]	3.79	H
His-227	[ND1]	Ser-69	[OG]	2.83	H
Ala-183	[N]	Ala-70	[O]	3.32	H
Ala-183	[O]	Val-71	[N]	3.59	H
Ala-181	[O]	Cys-72	[SG]	3.80	H
His-178	[ND1]	Cys-101	[O]	3.65	H

The central MMP-binding epitope of TIMP-2, formed by the N-terminal segment and disulfide-linked C-connector loop, fills the entire substrate binding cleft of the catalytic domain of MMP-10 (**Fig. 3b**), in a manner similar to that described for the previously solved structures of MMP-13/TIMP-2 (PDB ID: 2E2D) [28] and MT1-MMP/TIMP-2 (PDB ID: 1BQQ) [27]. Residues Pro_67_-Ser_68_-Ser_69_-Ala_70_-Val_71_-Cys_72_ occupy the non-primed side of the binding cleft, to the left of the catalytic zinc ion in the standard metallopeptidase orientation [46]. Residues Cys_1_-Ser_2_-Cys_3_-Ser_4_-Pro_5_ fill the primed side of the binding site to the right of the zinc ion, forming main-chain hydrogen bonds to the protease (**Table 2**). The carbonyl oxygen and amino group of N-terminal residue Cys-1, expected here to be in the uncharged state [47], coordinate the catalytic zinc ion, while the side chain of Ser-2 is deeply embedded in the S_1_′ specificity subsite (**Fig. 3b**).

To the upper left of the substrate binding cleft, the long AB loop of TIMP-2 makes contact with a groove between the βIV-βV loop and the S-loop of MMP-10 (**Fig. 3a, 3c**). While substantial surface area is buried here, the two molecules do not appear to have optimal shape complementarity or many specific stabilizing interactions in this region. In particular, Tyr-36 at the tip of the loop does not seem to interact very closely with MMP-10, unlike the complex with MT1-MMP, where Tyr-36 is buried in a groove of the MMP, forming an interfacial H-bond (PDB ID: 1BQQ) [27], and acts as a critical specificity determinant [48]. The side chain of TIMP-2 residue Ile-40 forms hydrophobic interactions with MMP-10 residues Phe-170 and Tyr-171 in the S-loop and with Leu-190 in the βIV-βV loop of the enzyme (**Fig. 3c**). Additionally, backbone-to-side chain hydrogen bonds formed between TIMP-2 Lys-41 and MMP-10 Tyr-171 and between TIMP-2 Arg-42 and MMP-10 Phe-170 stabilize the molecular interaction in this region (**Fig. 3c**).

Close interactions between the MMP-10cd and the C-terminal domain of TIMP-2 are quite minimal in this structure. TIMP-2 residues Arg-132, Pro-134, and Met-135 of the GH loop contact MMP-10 between the specificity loop and the βV-hB loop, while Trp-151, Lys-155, and Ile-157 of the TIMP-2 multiple turn loop make additional contacts with the MMP-10 specificity loop (**Fig. 3a, 3d**). No hydrogen bonds are identified in this region of the interface. The most notable favorable contacts in this region are aromatic interactions of TIMP-2 Trp-151 with Phe-242 of the MMP-10 specificity loop, featuring interatomic distances as short as 3.66 Å (Trp N_ε1_ to Phe C_ε1_), and cation-π interactions of the TIMP-2 Arg-132 side chain, which makes intermolecular contacts with MMP-10 aromatic residues Phe-242 (3.33 Å interatomic distance between Arg NH2 and Phe C_ε2_) and Tyr-239 (3.98 Å interatomic distance between Arg NH2 and Tyr C_ζ_) (**Fig. 3d**). The distances and geometries of these contacts are consistent with those found previously to contribute favorably to protein-protein and protein-peptide molecular recognition [49], [50].

### Structural determinants of binding in MMP-10 complexes with TIMP-1 and TIMP-2

**Figure 4:**
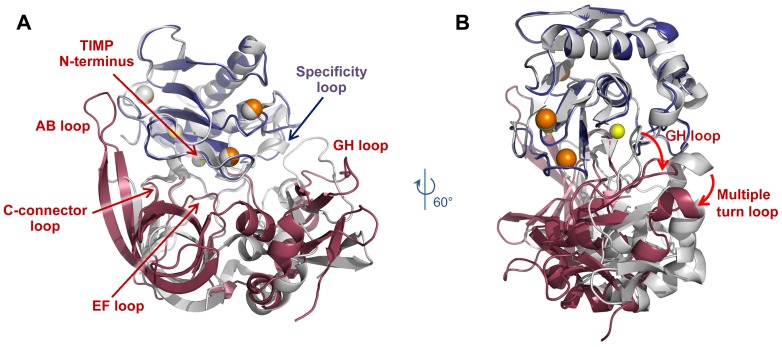
Comparison of MMP-10cd/TIMP-1 and MMP-10cd/TIMP-2 complexes. MMP-10cd/TIMP-2 molecules are shown in blue and raspberry, respectively, with MMP-10cd/TIMP-1 complex (PDB ID: 3V96) [24] shown in white; complexes are superposed based on C_α_ atoms of all MMP-10cd residues. (A) The long AB loop of TIMP-2 forms a much more extensive contact area with the MMP-10cd than is seen with TIMP-1, while the C-terminal loops of TIMP-2 form fewer contacts than in the complex with TIMP-1. (B) TIMP-2 is rotated by ∼21° around an axis centered on the catalytic zinc when compared with TIMP-1.

Our new structure of the MMP-10cd bound to TIMP-2 for the first time allows for comparison of complexes of a single MMP with different TIMPs. Human TIMP-1 and TIMP-2 share only 40% sequence identity, with amino acid differences concentrated in the AB loop, the C-connector-D loop, the multiple turn loop between helices h4a-h4b, and the flexible C-terminus. The TIMPs also vary in the lengths of several loops, the most striking difference being the AB loop, which in TIMP-2 is much longer and more exposed. Comparing the MMP-10cd/TIMP-2 complex (colored indigo/raspberry in **Fig. 4**) with that of our previously solved structure of MMP-10cd/TIMP-1 (colored white; PDB ID: 3V96) [24], we note that the long AB loop of TIMP-2 twists to form a more extended region of contact with the MMP-10cd, while the GH and multiple turn loops of the C-terminal domain are positioned further from the protease compared with those of TIMP-1 (**Fig. 4a, 4b**). These differences in positioning of peripheral loops are explained in part by a rotation in the orientation of TIMP-2 by ∼21° relative to the MMP-10cd (**Fig. 4b**).

In total, formation of the two complexes results in burial of a similar area of solvent accessible surface: 1247 and 1232 Å^2^ for MMP-10cd and TIMP-2, compared with 1173 and 1242 Å^2^ for MMP-10cd and TIMP-1. Despite the similarity in contact area, the change in solvation energy on binding is estimated using PISA [51] to favor complex formation by 12.5 kcal/mol for the MMP-10cd/TIMP-2 complex compared with 16.8 kcal/mol for the MMP-10cd/TIMP-1 complex. The greater predicted stability for the MMP-10cd/TIMP-1 complex is consistent with the experimental observation of modestly stronger binding affinity for this complex, with an inhibition constant (*K*_i_) of 1.1 nM compared with 5.7 nM for the MMP-10cd/TIMP-2 complex [24]. A generally poorer alignment of complementary features in the MMP-10cd/TIMP-2 complex contributes to the differences in binding affinity; 6 of 19 interfacial H-bonds identified by PISA in the MMP-10cd/TIMP-2 complex were of a distance >3.5 Å, compared with 1 of 19 H-bonds in the MMP-10cd/TIMP-1 complex (see Table 2; compare with Table 3 of reference [24]).

**Table 3:** Buried Surface Area (BSA).

PDB ID	Complex	N-TIMP/MMP BSA (Å^2^)	N-TIMP/MMP ΔG (kcal/mol)†	C-TIMP/MMP BSA (Å^2^)	C-TIMP/MMP ΔG (kcal/mol)†
**4ILW**	MMP-10cd/TIMP-2	1131.5/1137.2	−11.7	127.7/122.8	−2.7
**3V96**	MMP-10cd/TIMP-1	892.1/793.5	−12.0	405.5/415.5	−4.1
**1UEA**	MMP-3cd/TIMP-1	1025.3/939.5	−17.7	416.6/433.4	−6.8
**1BQQ**	MT1-MMPcd/TIMP-2	1136.0/1049.2	−15.6	547.0/627.2	−3.0
**2E2D**	MMP-13cd/TIMP-2	949.6/850.1	−14.2	356.4/342.8	−2.0

† ΔG refers to changes in solvation energy on complex formation.

Comparison of the MMP-10cd/TIMP-2 and MMP-10cd/TIMP-1 complexes also shows that despite substantial structural differences between the TIMPs, most of the flexible loops of the MMP-10cd are locked into nearly indistinguishable conformations upon TIMP binding (**Fig. 4a**). Superposition of MMP-10cd molecules reveals an overall r.m.s.d. of 0.233 Å for equivalent Cα atoms between the two structures. The similarity of MMP-10cd conformations in TIMP-1 and TIMP-2-bound structures is somewhat surprising, given the striking variability in loop structures revealed by MMPs bound to different small molecule inhibitors and the loop mobility evident in NMR solution structures of MMP catalytic domains [52], [53]. If stabilization of a common bound MMP conformation proves to be a general property of TIMPs, this phenomenon may facilitate structure-based design of engineered TIMPs optimized for selectivity toward individual MMPs, an approach that has proven very challenging for small molecule MMP inhibitors [53].

The single region of the MMP-10cd that does show conformational differences between TIMP-1 and TIMP-2-bound structures is found in the specificity loop. While residues 244-249 of this flexible loop take on highly similar conformations upon binding to either TIMP-1 or TIMP-2, residues 239–243 are found in differing conformations in the two TIMP-bound structures (**Fig. 4a**). Notably, this deviating segment abuts the C-terminal domain of the TIMP, and forms differential contacts with TIMP-1 and TIMP-2. Whereas the only significant interactions with the TIMP-2 GH and multiple turn loops are the aromatic and cation-π interactions formed with the TIMP-2 Arg-132 and Trp-151 side chains (**Fig. 3d**), the MMP-10cd can form more extensive interactions with the GH and multiple turn loops of TIMP-1, as we have described previously [24].

### Global conservation and diversity of MMP-TIMP binding determinants

**Figure 5:**
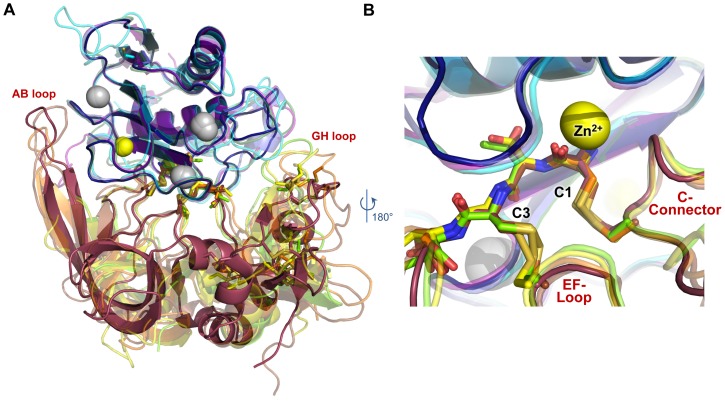
MMP/TIMP complexes feature conserved core interactions but highly diverse peripheral interactions. MMP-10cd/TIMP-2 (indigo/raspberry) is superposed with four different MMPcd/TIMP structures based on C_α_ atoms of all corresponding MMP residues: MMP-10cd/TIMP-1 (slate/chartreuse; PDB ID: 3V96) [24], MMP-3cd/TIMP-1 (purple/yellow; PDB ID: 1UEA) [26], MT1-MMPcd/TIMP-2 (cyan/brown; PDB ID: 1BQQ) [27], and MMP-13cd/TIMP-2 (forest/orange; PDB ID: 2E2D) [28]. (A) Positioning of peripheral TIMP loops including the AB and GH loops relative to the MMP show wide variability. (B) In the MMP active site, backbone positioning of TIMP residues 1–4 and the C-connector loop are nearly identical.

In **Figure 5**, comparison of the MMP-10cd/TIMP-2 (indigo/raspberry) and MMP-10cd/TIMP-1 (slate/chartreuse) structures with previously reported structures of MMP-3cd/TIMP-1 (purple/yellow; PDB ID: 1UEA) [26], MT1-MMPcd/TIMP-2 (cyan/brown; PDB ID: 1BQQ) [27], and MMP-13cd/TIMP-2 (forest/orange; PDB ID: 2E2D) [28] enables us to distinguish those contacts that are universally conserved in MMP-TIMP interactions from those that are unique to specific MMP/TIMP complexes. Superposition of all five complexes reveals extreme diversity in the positioning of peripheral loops of both TIMP domains relative to the MMP catalytic domains (**Fig. 5a**). In particular, the involvement of AB, GH, and multiple turn loops of TIMPs in interactions with MMP catalytic domains ranges from extensive to almost inconsequential. On the other hand, residues 1–4 of the TIMP N-terminal segment, along with five residues of the C-connector loop (residues 68–72 of TIMP-2 or 66–70 of TIMP-1) assume nearly identical conformations in the five MMP-TIMP complexes, and make interactions with the MMP catalytic domains that are highly structurally conserved (**Fig. 5b**).

**Figure 6:**
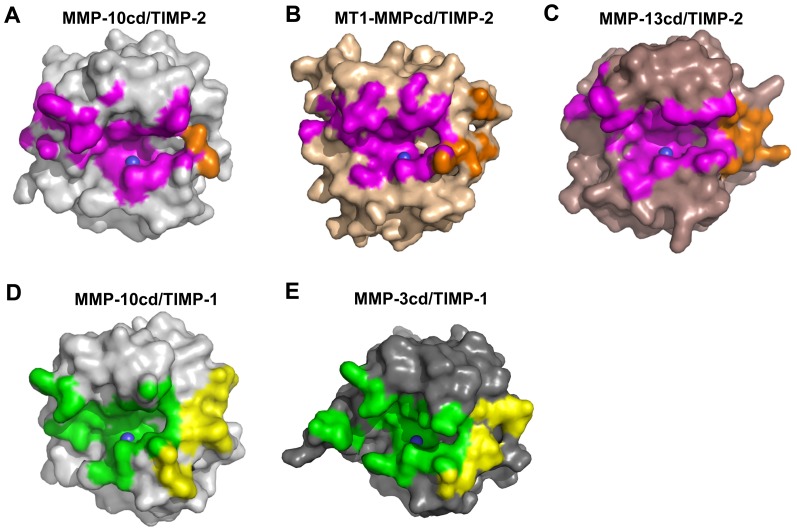
Diversity of MMP contact surfaces interacting with TIMP N- and C-terminal domains. MMPs are shown in the standard frontal orientation, with catalytic zinc shown as a blue sphere. (A) MMP-10cd/TIMP-2: MMP-10cd surface is shown in grey with footprint of the surface buried by TIMP-2 N-terminal domain in magenta and C-terminal domain in orange. (B) MT1-MMPcd/TIMP-2 (PDB ID: 1BQQ) [27]: MT1-MMPcd surface is shown in wheat with footprint of the surface buried by TIMP-2 N-terminal domain in magenta and C-terminal domain in orange. (C) MMP-13cd/TIMP-2 (PDB ID: 2E2D) [28]: MMP-13cd surface is shown in brown with footprint of the surface buried by TIMP-2 N-terminal domain in magenta and C-terminal domain in orange. (D) MMP-10cd/TIMP-1 (PDB ID: 3V96) [24]: MMP-10cd surface is shown in grey with footprint of the surface buried by TIMP-1 N-terminal domain in green and C-terminal domain in yellow. (E) MMP-3cd/TIMP-1 (PDB ID: 1UEA) [26]: MMP-3cd surface is shown in dark grey with footprint of the surface buried by TIMP-1 N-terminal domain in green and C-terminal domain in yellow. The MMP contact surfaces shown are for atoms within 4.5 Å of the TIMP; figures were generated using PYMOL.

The contributions of TIMP N- and C-terminal domains to interface contacts and complex stability vary considerably between the different complexes. For example, we see that the proportion of the MMP/TIMP intermolecular contact surface that is contributed by the C-terminal domain of the TIMP ranges from very minimal (less that 10% of total buried surface area for the MMP-10cd/TIMP-2 complex) to more extensive (greater than 30% of total buried surface area for the MMP-10cd/TIMP-1, MMP-3cd/TIMP-1, and MT1-MMPcd/TIMP-2 complexes) (**Table 3**). These differences can be appreciated visually in examining the footprints of TIMP contact regions on the MMP catalytic domain molecular surfaces (**Fig. 6**). Contributions to molecular complex stability span a similarly broad spectrum, with significantly greater involvement of the C-terminal domain in the complexes of TIMP-1 with MMP-3cd and MMP-10cd, compared with the complexes of TIMP-2 with MMP-10cd, MT1-MMPcd, and MMP-13cd (**Table 3**).

From the vantage point offered by comparative structural analysis, the TIMP N-terminus and C-connector loop form an integrated epitope targeting the MMP active site in a conserved fashion, while the peripheral loop contacts can be viewed as a separate category of adventitious “exosite” interactions. These exosite contacts are distinct to each complex and result from induced fit of flexible TIMP loops to the varying steric and electrostatic surfaces of different MMPs. It is very likely that the peripheral loops of TIMPs can exploit natural regulatory sites on the surface of MMP catalytic domains [54], forming opportunistic interactions. Intriguingly, the multiple turn loop and N-terminal strand residues 3–5 of both TIMP-1 and TIMP-2 in the complexes with MMPs are positioned near to a recently identified allosteric regulatory site of MMP-12, while the long AB loop of TIMP-2 is capable of forming contacts near to a regulatory site in MT1-MMP [54]. We would speculate, given the diversity of regulatory binding sites computationally predicted to be present in different MMP catalytic domains [54], that the four human TIMPs will differ substantially in their ability to exploit specific sites, leading to differences of affinity and binding kinetics among MMP-TIMP pairs that may fine-tune each TIMP molecule for distinct regulatory roles.

## Conclusions

Our structures of the MMP-10cd complexes with TIMP-1 and TIMP-2, in concert with the several previously reported MMP/TIMP structures, allow us to identify core interactions that are universally conserved at the heart of MMP-TIMP complexes, involving four residues at the TIMP N-terminus and five residues of the C-connector loop. We also identify a second category of adventitious interactions in which flexible peripheral loops of TIMPs adapt to form unique interactions in different complexes; mediators of these interactions include the AB loop, GH loop, and multiple turn loop. We observe that the N-terminal segment and CD loop form an integrated epitope targeting the MMP active site in a conserved fashion, while the peripheral loop and domain contacts can be viewed as separate “exosite” interactions. Our data suggest that these peripheral interactions have the potential to significantly enhance MMP/TIMP binding, although they do not appear to be well optimized to do so in the MMP-10/TIMP-2 complex. A potential consequence of this limited molecular complementarity, in an *in vivo* setting characterized by multiple MMPs competing for a limited pool of TIMPs, may be an excess of residual active MMP-10, a situation that may contribute to pathogenesis in diseases such as lung cancer [16], [17], [18]. One caveat to this interpretion is that our binding and structural analyses have thus far been limited to the truncated MMP-10 catalytic domain lacking the hemopexin domain. Although the MMP-10 hemopexin domain has not been reported possess independent affinity to any TIMPs, it is possible that this domain may subtly alter affinity through unrecognized favorable or deleterious interactions; further studies will be required to resolve this point.

The natural variability in TIMP/MMP binding preferences points to the potential for developing more highly selective “designer TIMPs” via protein engineering of natural TIMP scaffolds [55], [56]. Successes thus far in engineering TIMPs for enhanced selectivity have focused primarily on residues of the central inhibitory epitope [56], [57], [58], although selected mutations in the AB loop have also been found to modulate the affinity of TIMP-2 toward MT1-MMP [48] and of TIMP-4 toward the tumor necrosis factor-α-converting enzyme [59]. Based on our structural analyses, we suggest that peripheral epitopes, presented not only by the AB loop but also by the C-terminal domain of the TIMP scaffold, might be more intensively targeted for optimization to enhance inhibitor selectivity toward individual MMPs. Such efforts could take advantage of diversity library screening and directed evolution, such as the phage display approach recently successful in identifying an MMP-1-selective variant of TIMP-2 [60]. Ultimately, continued efforts to understand and exploit the structural basis of molecular recognition of MMPs by TIMPs may facilitate development of new MMP-directed probes and therapeutics, taking advantage of emerging concepts for delivery of TIMP-based drugs and probes *in vivo* [61], [62], [63].
